# A novel format for recombinant antibody-interleukin-2 fusion proteins exhibits superior tumor-targeting properties *in vivo*

**DOI:** 10.18632/oncotarget.27726

**Published:** 2020-10-13

**Authors:** Tiziano Ongaro, Baptiste Gouyou, Marco Stringhini, Riccardo Corbellari, Dario Neri, Alessandra Villa

**Affiliations:** ^1^Philochem AG, Otelfingen, Switzerland; ^2^University School for Advanced Studies IUSS Pavia, Pavia, Italy; ^3^Department of Chemistry and Applied Biosciences, Swiss Federal Institute of Technology, ETH Zürich, Zürich, Switzerland; ^4^University of Trento, CiBIO, Department of Cellular, Computational and Integrative Biology, Trento, Italy

**Keywords:** tumor targeting, fibronectin, immunocytokines, interleukin-2, protein engineering

## Abstract

The targeted delivery of interleukin-2 to the tumor is gaining attention as an avenue to potentiate the action of T and NK cells at the site of disease. We have previously described the fusion of the L19 antibody, specific to the EDB domain of fibronectin, with human interleukin-2, using a non-covalent homodimeric diabody format. Here, we describe four novel formats for the L19-IL2 fusion, featuring different arrangements of antibody and IL2. A comparative quantitative biodistribution analysis in tumor-bearing mice using radioiodinated proteins revealed that the novel format (L19L19-IL2, with the antibody in single-chain diabody format) exhibited the best biodistribution results. *In vitro* assays on peripheral blood mononuclear cells showed a decrease activation of regulatory T cells when single IL2 domain was used. *In vivo*, both L19-IL2 and L19L19-IL2 inhibited tumor growth in immunocompetent mouse models of cancer. T-cell analysis revealed similar levels of CD4^+^ and FoxP3^+^ cells, with an expansion of the CD8^+^ T cell in mice treated with L19-IL2 and L19L19-IL2. The percentage of CD4^+^ regulatory T cells was markedly decreased with L19L19-IL2 combined with a mouse-specific PD-1 blocker. Collectively, these data indicate that the new L19L19-IL2 format exhibits favorable tumor-homing properties and mediates a potent anti-cancer activity *in vivo*.

## INTRODUCTION

There is a growing interest in the use of immunotherapy approaches for the treatment of cancer, which has been promoted by the clinical results obtained against various types of malignancies using anti-PD-1 and anti-PD-L1 antibodies [1–4].

Certain pro-inflammatory cytokines (e.g., interleukin-2) may provide a complementary anti-cancer activity and may be ideal combination partners, in addition not only to immune checkpoint inhibitors [5, 6], but also to radiation [7, 8] and cytotoxic agents [9]. IL2 was approved by FDA for metastatic melanoma patients in 1998, based on the observation that a small proportion of subjects enjoyed durable complete responses [10]. However, treatments in these dose regimens often lead to severe side effects, including fever and chills, hypotension, fatigue and vascular leak syndrome, which in extreme cases can lead to organ failure with lethal consequences [11]. Therefore, high dosage treatments are usually reserved to young and physically fit patients only. For this reason, current research efforts aim at the development of IL2 therapeutics with improved therapeutic index. Pegylated forms of IL2 have shown superior properties in mouse models of cancer [12–14] and encouraging results in patients as single agents [15] or in combination with anti-PD-1 antibodies [16, 17]. The masking of IL2 epitopes using antibodies allowed the selective activation of lymphocytes sub-populations, improving immune-stimulatory activity [18]. Similar results were achieved by *de novo* computationally designed IL2 [19].

Various groups [20–22], including our own [23–25], have worked on the concept of fusing the IL2 moiety with suitable tumor-homing antibodies, in order to concentrate the payload at the site of disease, helping spare normal tissues [23]. While some researchers have fused the IL2 moiety at the C-terminal extremity of the heavy chain [21] or of the light chain [26] of antibodies in full IgG format, we have preferred to use antibody fragments, as they may exhibit more favorable tumor: organ ratios and as those products may clear more rapidly from circulation, thus avoiding prolonged cytokine-related toxicity.

L19 is a fully human, clinical grade antibody that targets the alternatively spliced EDB domain of fibronectin. It reacts with identical affinity toward the human and murine EDB containing fibronectin, allowing an easier translation of experiment in human [27]. EDB is a 91-amino acid domain which is identical in mouse, rat, rabbit dog and man and can be inserted by alternative splicing into the fibronectin molecule [28]. It is expressed during active tissue remodeling and therefore represents an appealing target for the selective delivery of payloads at the tumor site, since it accumulates around neovasculature structures in tumors and is virtually undetectable in healthy organs (exception made for some female reproductive organs) [29, 30]. The value of EDB as target for pharmacodelivery applications has been proven both in preclinical animal models and in patients by quantitative biodistribution studies [31–33], imaging studies with nuclear medicine techniques [34] and in the pharmacodelivery of radionuclides [35].

We have previously developed IL2-based immunocytokines composed of non-covalent homodimeric scFv fragments delivery vehicle. One of these products, L19-IL2 is currently being evaluated in phase II clinical trials as single immunostimulatory agent [NCT02957019] and phase III in combination with targeted TNF for the of patients with fully resectable stage IIIB, C melanoma [NCT02938299]. In this article, we have investigated four novel formats for the fusion of IL2 to recombinant antibody versions of the L19 antibody. We have performed a comparative biodistribution analysis in tumor-bearing mice, as well as an evaluation of pharmaceutical properties *in vitro* and in three different immunocompetent mouse models of cancer. One of the new products (termed L19L19-IL2) featured the L19 antibody in single-chain diabody format [36] and exhibited favorable properties in various types of functional analysis. L19L19-IL2 may thus represent a valuable alternative to L19-IL2 for future clinical development programs.

## RESULTS

### Production and initial characterization of L19-IL2 and of four novel fusion proteins

**Figure 1:**
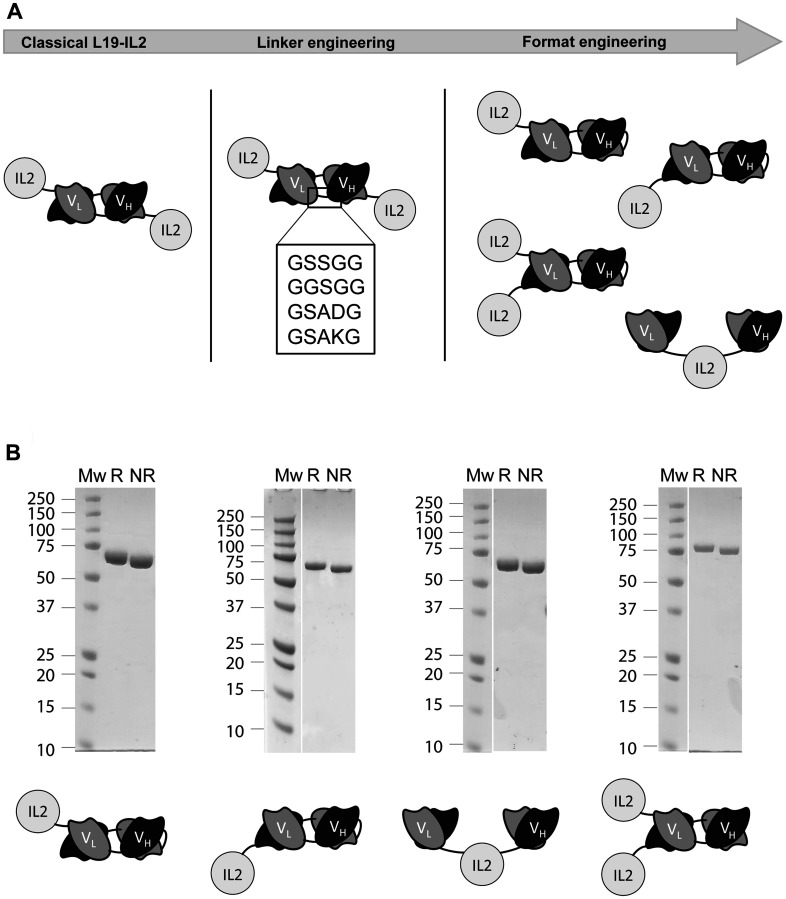
(**A**) Graphic representation of the all L19-IL2 fusion candidates comprising different VH-VL domain linker sequences (linker engineering), different arrangements between the IL2 and the L19 domains and different number of payloads (format engineering). (**B**) SDS-PAGE characterization of all explored formats.

Eight novel immunocytokines comprising the variable heavy (VH) chain and variable light (VL) chain domains of the L19 antibody and an IL2 payload were generated. At first, four candidates bearing different linkers between the VH and VL were cloned. The L19-IL2 immunocytokines were designed to comprise different linker sequences between the VH and VL domains in diabody format (neutral, positively charged and negatively charged). Subsequently, four more immunocytokines with novel formats were produced. These differed among each other for the spatial arrangement between the antigen binding fragment and the cytokine and, more importantly, for the number IL2 moieties (either one or two) (Figure 1A). All immunocytokines were characterized *in vitro* by SDS PAGE (Figure 1B) where a clean band was observable corresponding to the expected molecular weight. Production yields and characterization including of size exclusion chromatography, BIAcore and immunofluorescence are reported in Supplementary Figure 1. All the candidates showed only minor differences when characterized *in vitro*.

**Figure 2:**
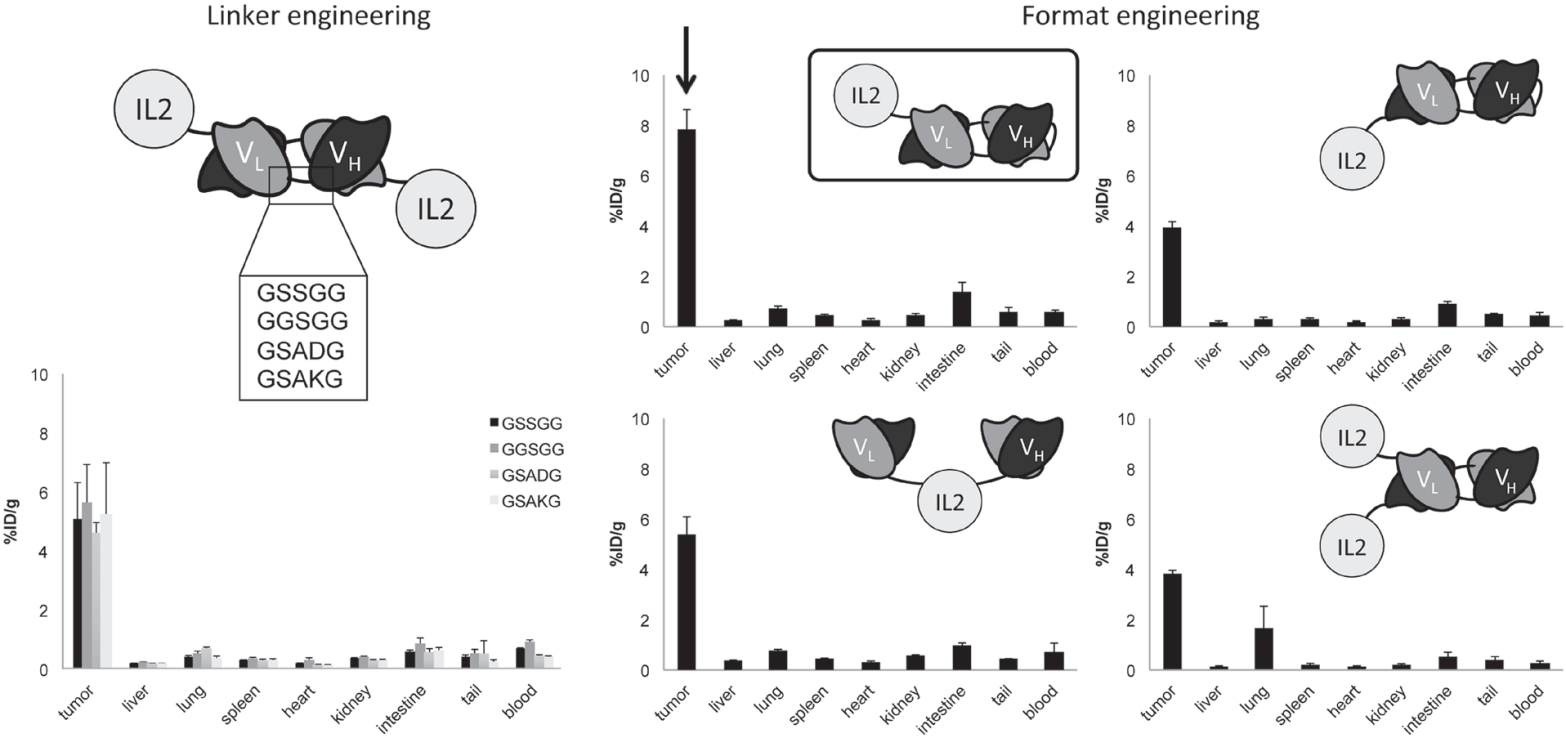
*In vivo* comparative quantitative biodistribution studies performed in immunocompetent mice bearing F9 teratocarcinoma tumors (*n* = 3 per group), using radioiodinated protein preparation. Data represent means of percentage of injected dose per gram (%ID/g) values in tumor, blood and normal organs ± SEM at 24 hours after intravenous injection.

*In vivo* biodistribution studies were performed in F9 tumor-bearing mice using radioiodinated immunocytokine preparations. One immunocytokine format, composed by two L19 scFv domains in tandem diabody format linked to an IL2 payload at the C-terminus (named L19L19-IL2), had significantly superior tumor targeting properties, as reflected by the highest percentage of injected dose-per gram of the immunocytokine in the tumor, compared to the other tested formats (except for the GGSGG candidate where L19L19-IL2 had a higher accumulation but not significantly superior). Statistical analysis are reported in Supplementary Table 3A. Specifically, L19L19-IL2 showed an accumulation of about 7.7% ID/g in the tumor and a tumor-to-blood ratio of about 13, while the other immunocytokines reached values of approximatively 5% ID/g in the tumor (Figure 2, Supplementary Table 3B). However, it has to be considered that the biodistribution experiments evaluated the quantity of immunocytokine at the tumor site and not the quantity of IL2. In light of these results, we decided to further characterize the L19L19-IL2 format and benchmark it with our clinical grade L19-IL2 protein.

The monomeric nature of the new format could be an advantage in reducing the avidity effect of the regulatory T cells, thus reducing the inhibition of the anti-tumor response. We therefore compared the proliferation effect of the new monomeric L19L19-IL2 with the dimeric L19-IL2 on immune cell population of PBMCs.

**Figure 3:**
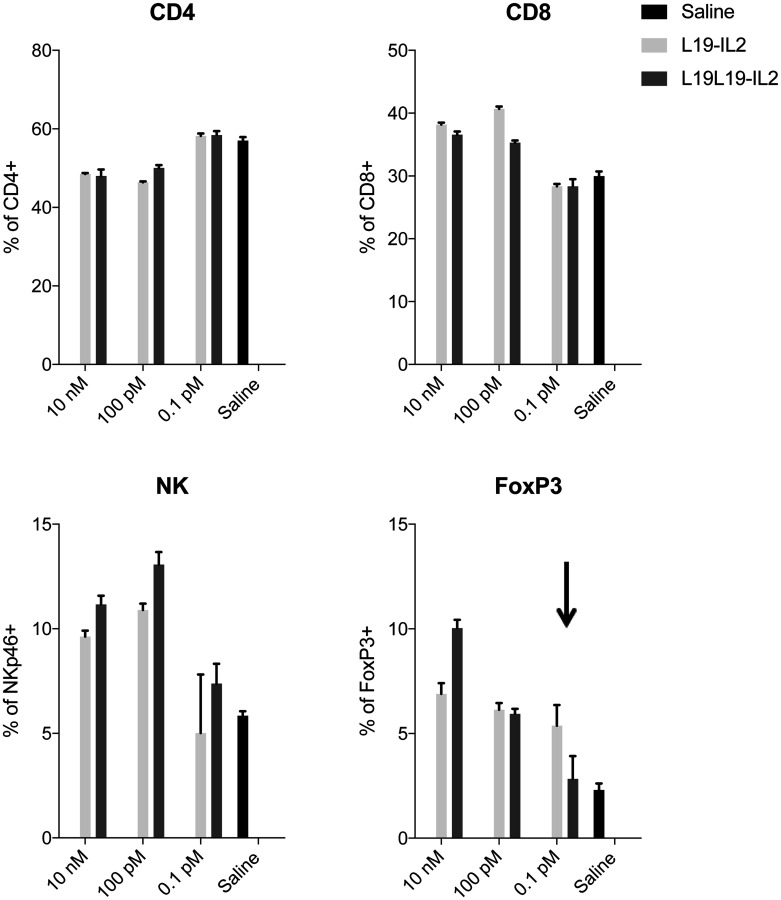
PBMCs proliferation assay. Expansion of CD4^+^, CD8^+^, NKp46^+^ and FoxP3^+^ cells was determined by antibody staining followed by flow cytometry analysis. Data represent means ± SEM, (*n* = 3).

Human PBMCs were isolated and incubated with different concentrations of the two candidates and a saline as reference for 5 days. Afterwards, cellular proliferation and T cells subpopulation were analyzed by FACS. A higher (but still comparable) percentage of FoxP3^+^ lymphocytes could be observed in PBMCs incubated with L19L19-IL2 at 10 nM concentration. On the other hand, a lower percentage of FoxP3^+^ was notable in PBMCs incubated with the monomeric L19L19-IL2 at the lowest concentration (Figure 3). This result support the hypothesis that the new format might be less prone to stimulate regulatory T cells activity compared to the dimeric L19-IL2 at lower dose. There were no significant differences in the other subpopulation percentages.

### Comparative evaluation of the therapeutic properties of L19-IL2 and L19L19-IL2 in three different mouse models of cancer

**Figure 4:**
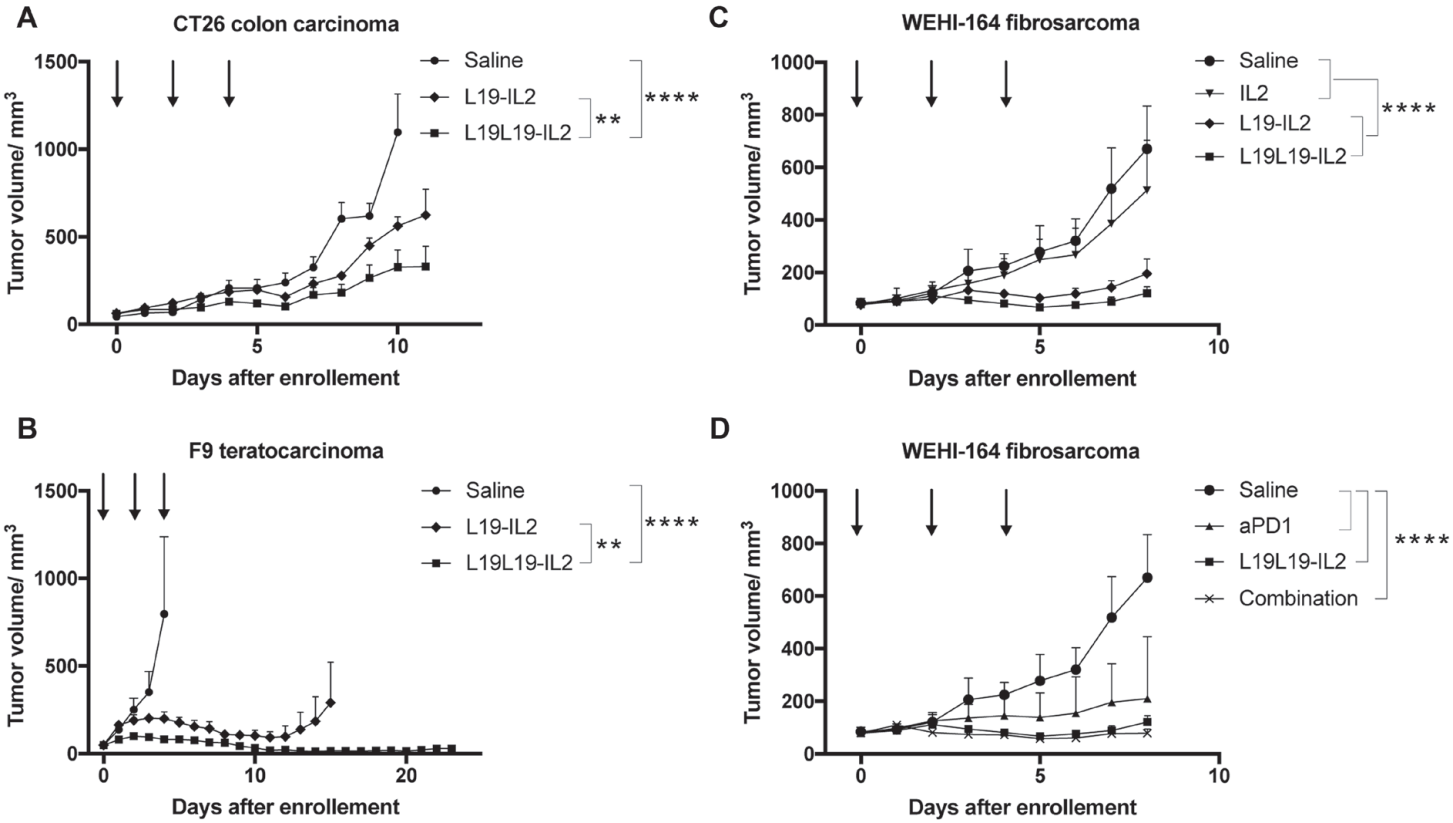
Tumor therapy experiments in immune competent mice. Treatments started when tumors reached a size of approximatively 80 mm^3^. Fusion proteins were administered every second day for three times. (**A**) Therapy with L19-IL2 and L19L19-IL2 in BALB/c mice bearing CT26 colon carcinoma tumors. The fusion proteins were administered at 50 μg and 82.5 μg in order to have equimolarity. (**B**) Therapy with L19-IL2 and L19L19-IL2 in 129/ScEv mice bearing F9 teratocarcinoma tumors. 100 μg of L19-IL2 and 165 μg of L19L19-IL2 were administered. (**C**) Therapy in BALB/c mice bearing WEHI-164 fibrosarcoma with therapeutic proteins administered as single agents. 40 μg of recombinant IL2, 100 μg of L19-IL2 and 165 μg of L19L19-IL2 were injected. (**D**) The same setting was evaluated in combination with the anti-PD-1 checkpoint inhibitor. 200 μg of anti-PD-1, 165 μg of L19L19-IL2 and a combination of the two were administered. Statistical significances were determined with a regular two-way ANOVA test with Bonferroni post-test correction. Data represent means ± SEM. ^*^ = *p* < 0.05, ^**^ = *p* < 0.01, ^****^ = *p* < 0.0001.

The *in vivo* performance of the new immunocytokine L19L19-IL2 was first evaluated in immunocompetent mice bearing subcutaneous CT26 colorectal carcinomas. The treatment started when the subcutaneously grafted tumors reached a size of approximatively 80 mm^3^. The new tandem diabody format was compared to the benchmark diabody format [37, 38] and to a saline. Immunocytokines were administered at equimolarity of IL2, meaning 50 μg of L19-IL2 diabody and 82.5 μg of L19L19-IL2 tandem diabody. Injections were done every second day for three times. L19-IL2 and L19L19-IL2 displayed comparable therapeutic activity, with the new format being slightly more effective in reducing the tumor growth (Figure 4). Body weight changes are reported in Supplementary Figure 4. Plasma collected 24 hours after the third injection showed that both immunocytokines successfully increased the level of plasmatic TNF-α and decreased the level of TGF-β. Moreover, a significant increase of interferon γ in the plasma of mice treated with L19L19-IL2 was notable. Overall, the level of IL2 in the plasma was < 2 pg/ml, confirming the good targeting properties of the candidates (Supplementary Figure 2). Endotoxin level of the injected proteins was measured and resulted to be far below the recommended daily limit [39] (Supplementary Figure 3).

A second study was performed in a F9 teratocarcinoma model in 129 Sv mice. Exponentially growing tumor cells where injected in the right flank of the mice and treatment was started when tumors reached an average size of about 80 mm^3^. Since no significant toxicity was observed in the CT26 model, we chose to increase the dose to 100 μg of L19-IL2 and 165 μg of L19L19-IL2. Both L19-IL2 and L19L19-IL2 showed therapeutic activity in slowing the tumor progression down, with the new format that achieved a stronger and longer lasting response (Figure 4). Better performances of L19L19-IL2 could also be observed in the number of complete remissions: 3 for L19L19-IL2 compared to 2 for L19-IL2. No significant changes in the serum cytokine were observed in this experiment (Supplementary Figure 2B).

A last *in vivo* experiment was conducted in WEHI-164 model in BALB/c mice. The WEHI-164 model had previously been reported to respond well to immune check point inhibitors, both as monotherapy and combination therapy [6]. Therefore, a treatment group with the combination of L19L19-IL2 and anti-PD-1 was included in this experiment. Exponentially growing WEHI-164 fibrosarcoma cells were injected in BALB/c mice and treatment started as tumors reached about 80 mm^3^ of average size. Also in this setting, L19L19-IL2 showed slightly better performances than L19-IL2 in diabody format. Both formats displayed a potent tumor growth inhibition compared to the saline control and to the untargeted recombinant interleukin-2. As expected, the checkpoint inhibitor anti PD-1 showed a significant tumor growth retardation. The response obtained by administration of anti-PD-1 and L19L19-IL2, however, was not significantly better than the monotherapy with L19L19-IL2 in term of tumor growth inhibition (Figure 4). A durable response could be observed in 4 out of 5 mice treated with the combination. In these mice, no tumor growth could be observed up to 43 days after the therapy start.

**Figure 5:**
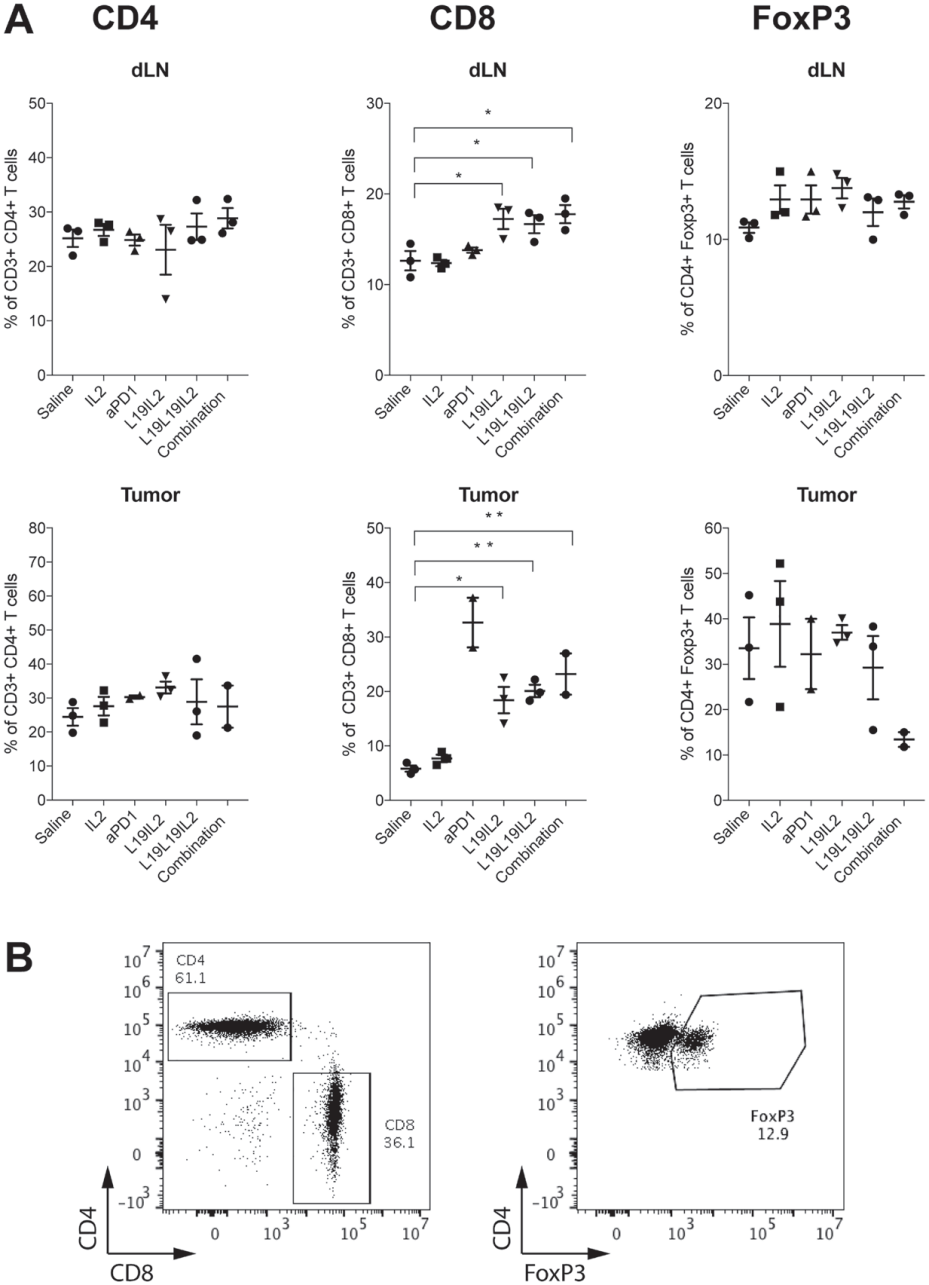
(**A**) Immunophenotypic analysis of lymphocyte in tumor and in tumor draining lymph node (dLN) of treated mice. Individual plots show the percentage of CD4^+^ T cells, CD8^+^ T cells and CD4^+^ FoxP3^+^ T cells in draining lymph nodes on mice from the different therapy groups (*n* = 2–3). Statistical significances were determined with a regular one-way ANOVA test with Bonferroni post-test correction. Data represent means ± SEM. ^*^ = *p* < 0.05, ^**^ = *p* < 0.01. (**B**) Representative FACS plots of CD4^+^ T cell, CD8^+^ T cell (left) and CD4^+^ FoxP3^+^ T cell subsets (right).

To better understand the mechanism behind the anti-cancer response in the various treatment groups, we analyzed the T cells subpopulations in draining lymph nodes and in the tumors of treated WEHI–164 bearing mice. In total, four staining were performed to differentiate (i) CD3^+^, CD4^+^, CD8^+^ and NK cells, (ii) CD44^+^, CD62L^+^, CD8^+^ and AH1^+^ cells, (iii) CD4^+^, CD8^+^, PD-1^+^ and TIM3^+^ cells and (iv) CD4^+^, FoxP3^+^ cells. A significant increase in the percentage of CD8^+^ T cells could be observed both in the lymph nodes and in the tumors of the mice treated with L19-IL2, L19L19-IL2 and L19L19-IL2 in combination with anti-PD-1 but not in the mice treated with non-targeted recombinant IL2. Mice treated with anti-PD-1 alone showed a significant increase of CD8^+^ T cells only in the tumor. A decrease of CD4^+^PD-1^+^ cells in the lymph node of mice treated with L19L19-IL2 alone or in combination with anti-PD-1 could be observed. Importantly, there was a decreasing trend of CD4^+^FoxP3^+^ in the tumors of mice treated with L19L19-IL2 with anti-PD-1 (Figure 5).

## DISCUSSION

L19-IL2 is an immunocytokine featuring the L19 antibody fused to the cytokine IL2 payload, which is currently being investigated with encouraging results for the treatment of metastatic melanoma in phase II and phase III clinical trials, in combination with targeted TNF. In this study, we have investigated the therapeutic potential of four novel formats of immunocytokines based on the L19 antibody in single-chain diabody format and an IL2 payload.

*In vitro* characterization by multiple biochemical and immunological techniques showed that the various L19-IL2 fusion proteins were homogeneous and exhibited comparable binding properties. However, one of the newly developed formats (L19L19-IL2) showed an improved tumor uptake in quantitative biodistribution studies. *In vitro*, L19L19-IL2 showed a slightly decreased activation of regulatory T cells at low concentration and a higher percentage at 10 nM concentration. This effect is compatible with the saturation of the IL2 receptor. It has been previously reported that opposite effects at low and high doses can be observed with cytokines [40, 41]. *In vivo,* both the conventional L19-IL2 format and the new L19L19-IL2 fusion protein revealed potent anticancer activity in three syngeneic mouse tumor models.

The observation that fusion proteins carrying two IL2 moieties may lead to a stronger stimulation of regulatory FoxP3^+^ T cells is of potential biomedical relevance. On one hand, low dose IL2 has been proposed as treatment option for certain conditions like graft-versus-host disease [42], systemic lupus erythematosus [43] and hepatitis C virus-induced vasculitis [44]. It is possible that the bivalent display of IL2 may lead to better therapeutics because of a higher binder avidity and improved T reg selectivity. On the other hand, it has been claimed that regulatory T cell activity may counteract cancer immunotherapeutics [45] and, for this reason, L19L19-IL2 may represent a promising monovalent alternative for anti-tumor development programs.

A durable complete regression in WEHI-164 tumor bearing mice was observed in mice treated with the combination of IL2 therapeutics with PD-1 blockade. Immune checkpoint inhibitors gained huge popularity due to their effectiveness in cancer therapy [4]. The use of IL2 with anti PD-1 is potentially a powerful combination strategy where IL2 stimulates natural killer cells and T lymphocytes and the PD-1 blockade hinders the immune cells exhaustion, which is typical of prolonged stimulation [46]. Recent studies have also described that engineered IL2 variants may well synergize with immune checkpoint inhibitors [47, 48]. The efficacy of this combination has been evaluated in clinical trials with encouraging results [NCT00058279, NCT01856023] [49, 50]. In immunocompetent BALB/c mice, the tumor rejection process is dominated by CD8^+^ T cells which recognize AH1, a retroviral antigen selectively expressed in cancer [51].

Recombinant IL2 has shown encouraging results in the treatment of metastatic melanoma and renal cell carcinoma. Unfortunately, because of severe side effects, optimal therapeutic regimens could be achieved only for young and healthy patients [11]. The targeting of IL2 at the site of disease by means of antibodies as vehicles has been demonstrated to be an elegant solution to increase its therapeutic index in both in pre-clinical [23, 24, 37] and in clinical research. In our study, we could observe a striking difference in the tumor growth retardation between mice treated with untargeted and mice treated with targeted IL2, where the last managed to achieve a stronger and prolonged effect.

L19-IL2 has been administered to more than 200 patients with different types of malignancies [52, 53]. The most advanced clinical studies include its use in combination with L19-TNF for the treatment of fully resectable stage IIIB, C melanoma [NCT02938299] and in combination with stereotactic ablative radiotherapy for the treatment of oligometastatic tumors [NCT03705403]. It is likely that the substitution of L19-IL2 with L19L19-IL2, ideally in combination with PD-1 blockade, may lead to a further increase of therapeutic activity. Phase II studies with L19-IL2 in patients with metastatic melanoma had shown that some patients enjoyed major durable responses with favorable safety profile [53, 54] but more than 50% of the subjects did not achieve a partial response. The use of improved molecular formats and of judiciously chosen combination partners will further expand the applicability and performance of IL2 therapeutics. L19L19-IL2 could represent a promising monovalent alternative to L19-IL2, which deserves to be clinically investigated, because of its favorable tumor homing properties and reduced activation of regulatory T cells.

## MATERIALS AND METHODS

### Cloning

#### Cloning of the L19 diabody-IL2 conjugate with a GSSGG VH-VL domain linker sequence

Firstly, the DNA fragment encoding the L19 diabody comprising the GSSGG VH-VL domain linker sequence was cloned by PCR-amplification of the L19 gene (Supplementary Table 1A). The used PCR primers are reported in the Supplementary Table 2A. For the L19 PCR, primers HindIIISIP and L19Linker were used. The DNA sequence encoding the IL2 was cloned from the IL2 gene (Supplementary Table 1B) using primers LinkerIL2 and IL2stopNot<. The two DNA fragments (L19 antibody and IL2) were assembled by means of PCR, amplified using primers HindIIISIP and IL2stopNotI, double digested with HindIII/NotI and cloned into a pcDNA 3.1 (+) vector.

#### Cloning of L19 diabody-IL2 conjugates with different VH-VL domain linker sequences

Three further diabody-IL2 conjugates were prepared by inserting linker sequences GGSGG, GSADG and GSAKG between the heavy and light chain variable domains of the L19 diabody-IL2 conjugate. The L19 diabody-IL2 conjugates were prepared by means of PCR assembly of a fragment “A” (encoding the L19 heavy chain variable domain), and a fragment “B” (encoding the L19 light chain variable domain and the IL2 payload). Fragments “A” and “B” were amplified from the L19 diabody-IL2 molecule comprising the GSSGG VH-VL domain linker using the primers listed in the Supplementary Table 2B.

The “A” and “B” fragments were then PCR-assembled, PCR-amplified and double digested with HindIII/NotI-HF and cloned into the double digested vector pcDNA 3.1 (+).

The resulting plasmids were amplified and used for cell transfection.

#### Cloning of the single-chain diabody C-terminal fusion protein (L19L19-IL2)

All the primers used for the cloning of the new formats of L19-IL2 are reported in the Supplementary Table 2C.

The C-terminal fusion protein (L19L19-IL2) coding sequence was generated using L19-IL2 as template.

The L19 gene was PCR amplified using primers LnkDP47> and L19G4S3<. The IL2 gene was amplified using primers G4S3IL2> and IL2StopNot<. The two intermediate fragments were PCR-assembled, PCR-amplified using primers HindLnk> and IL2StopNot<, double digested with HindIII/NotI-HF and cloned into a pcDNA 3.1 (+) vector (resulting into a pcDNA 3.1 vector containing the sequence HindIII (restriction site)-L19-IL2-NotI (restriction site)).

A second L19 gene was PCR amplified using primers NheLead> and L19Hind<, double digested by NheI/HindIII and inserted into the previously generated pcDNA 3.1 (+) vector containing the sequence HindIII (restriction site)-L19-IL2-NotI (restriction site), resulting into the full length L19L19-IL2.

The resulting plasmids were amplified and used for cell transfection.

#### Cloning of the single-chain diabody N-terminal fusion protein (IL2-L19L19)

The N-terminal fusion protein (IL2-L19L19) coding sequence has been generated using L19-IL2 as template.

The IL2 gene was amplified using primers LeadIL2> and IL2G4S3<. The L19 gene was PCR amplified from G4S3L19> and L19Hind<. The two intermediate fragments were PCR-assembled, PCR-amplified using primers NheLead> and L19Hind<, double digested with NheI/HindIII and cloned into a pcDNA 3.1 (+) vector (resulting into a pcDNA 3.1 (+) vector containing the sequence NheI (restriction site)-IL2-L19-HindIII (restriction site)).

A second L19 gene was PCR amplified a first time using primers LnkDP47> and L19StopNot< and a second time using primers HindLnk> and L19StopNot<. The DNA fragment was subsequently double digested using HindIII/Not-HF and inserted into the previously generated pcDNA 3.1 (+) vector containing the sequence NheI (restriction site)-IL2-L19-HindIII (restriction site), resulting in the full length IL2-L19L19.

The resulting plasmids were amplified and used for cell transfection.

#### Cloning of the L19 single-chain diabody with IL2 conjugated to its C- and N-termini (IL2-L19L19-IL2)

The IL2-L19L19-IL2 coding sequence was generated using the previously cloned L19L19-IL2 and IL2-L19L19 as starting material.

The vector pcDNA 3.1 (+) containing the sequence NheI (restriction site)-IL2-L19-HindIII (restriction site)-L19 was digested by NheI/HindIII in order to obtain the IL2-L19 DNA fragment. At the same time, the vector pcDNA 3.1 (+) containing the sequence NheI (restriction site)-L19- HindIII (restriction site)-L19-IL2 was digested by NheI/HindIII, in order to remove the first L19 moiety and replace it with NheI (restriction site)-IL2-L19-HindIII (restriction site). By doing so, the full length IL2-L19L19-IL2 was obtained.

#### Cloning of IL2 conjugated at its C-terminus and at its N-terminus to two single-chain diabodies (“Crab”)

The “Crab” immunocytokine L19-IL2-L19 coding sequence was generated using L19-IL2 as template.

A first DNA fragment was amplified using primers HindLead> and DP47G4S2.5<. A second DNA fragment was amplified using primers G4S2.5VL> and IL2G4S3Bam<. The two intermediate fragments were PCR-assembled, PCR-amplified using primers HindLead> and IL2G4S3Bam<, double digested with HindIII/BamHI and cloned into a pcDNA 3.1 (+) vector (resulting in the vector pcDNA 3.1 (+) containing HindIII (restriction site)-L19-IL2-BamHI (restriction site)).

A third DNA fragment was amplified using primers BamG4S3L19> and DP47G4S2.5<. A fourth DNA fragment was amplified using primers G4S2.5VL> and L19StopNot<. The two intermediate fragments were PCR-assembled, PCR-amplified using primers BamG4S3L19> and L19StopNot<, double digested with BamHI/NotI HF and cloned into the previously generated pcDNA 3.1 (+) vector containing the sequence HindIII (restriction site)-L19-IL2-BamHI (restriction site), resulting in the full length L19-IL2-L19 “Crab” molecule. The resulting plasmids were amplified and used for cell transfection.

### Expression and purification of L19-IL2 immunocytokines

#### Cell culture and transfection

Transfected CHO-S cells (Chinese Hamster Ovary; Invitrogen) were cultured in suspension in PowerCHO-2CD medium (Lonza), supplemented with Ultraglutamine-1 (Lonza), HT-supplement (Gibco) and an antibiotic-antimycotic (Gibco).

F9 teratocarcinoma cells, CT26 colon carcinoma cells and WEHI-164 fibrosarcoma cells (ATCC) were grown according to supplier’s protocol and kept in culture for no longer than 3 weeks. F9 cells were expanded and cultured in DMEM medium (Gibco) supplemented with 10% of fetal bovine serum and antibiotic-antimycotic (Gibco) while CT26 and WEHI-164 were expanded and cultured in RPMI1640 medium (Gibco) supplemented with 10% fetal bovine serum and antibiotic-antimycotic (Gibco).

#### Expression and purification of L19-IL2 immunocytokines

The different L19-IL2 candidates were expressed using transient gene expression in CHO-S cells. For 1 mL of production 4 × 10^6^ CHO-S cells in suspension were centrifuged and resuspended in 1 mL ProCHO4 medium (Lonza). 0.625 μg of plasmid DNAs followed by 2.5 μg polyethylene imine (PEI; 1 mg/mL solution in water at pH 7.0) per million cells were then added to the cells and gently mixed. The transfected cell cultures were incubated in a shaker incubator at 31°C for 6 days. The suspensions were then centrifuged at 4°C 4900 rpm for 30 min. Supernatant was harvested and filter by Nalgene Rapid-Flow Bottle Top Filter (Nalgene) and incubated for 2 h at room temperature and shaking conditions with protein A Sepharose. The resin was then loaded in a column support and thereafter washed with 200 mL of Buffer A (100 mM NaCl, 0.5 mM EDTA, 0.1% Tween 20 in PBS) and then with 200 mL of Buffer B (500 mM NaCl, 0.5 mM EDTA in PBS). The antibody product was eluted using 10–15 mL Glycine 100 mM and fractions of 1 mL were collected. OD at an absorbance of 280 nm (OD280) was measured and fractions containing protein (OD280 > 0.1 mg/mL) were pooled and loaded on SpectraPor dialysis membrane Mw 25,000 (Spectrum laboratories) and dialyzed in PBS o/n at 4°C.

### Characterization of the L19-IL2 immunocytokines

#### SDS-PAGE and size-exclusion chromatography

The purified immunocytokines were characterized by SDS-PAGE and size-exclusion chromatography. SDS-PAGE was performed with 10% gels (NP0302BOX, Invitrogen) under reducing and non-reducing conditions. Purified clones were analyzed by size-exclusion chromatography on a Superdex 200 increase 10/300 GL column on an ÄKTA FPLC (GE Healthcare, Amersham Biosciences).

#### Affinity measurements

Affinity measurements were performed by surface plasmon resonance using a BIAcore ×100 instrument (BIAcore, GE Healtchare) using a fibronectin 7B89 domain coated CM5 chip. Samples were injected as serial-dilutions, in a concentration range from 1 mM to 250 nM. Regeneration of the chip was performed using 10 mM HCl.

#### PBMCs proliferation assay

PBMCs were isolated from healthy donor by density gradient using Ficoll-Paque (GE Healthcare, 17-544-02). Briefly, 50 ml of blood were collected in Heparin tubes. Blood was diluted 1:1 with PBS pH 7.4, 2 mM EDTA and slowly added to 19 ml of Ficoll solution. Samples were centrifuged at 400 g for 40 minutes at room temperature with low acceleration and deceleration. Buffy-Coat was collected, washed with PBS pH 7.4, 2 mM EDTA and resuspended in X-Vivo 15 medium.

5 × 10^4^ PBMCs where incubated for 5 days at 37°C with either 10 nM, 100 pM or 0.1 pM of the fusion proteins L19-IL2 or L19L19-IL2 and saline solution as negative control. All the groups were done in triplicates. The fifth day, proliferation and T cells subpopulations were determined by flow cytometry. Fluorophore-conjugated antibodies against CD3 (clone HIT3a), CD4 (clone A161A1), CD8 (clone SK1), NKp46 (clone 9E2) and FoxP3 (clone 206D), as well as Zombie Red viability dye were all purchased from BioLegend.

PBMCs cells suspension were washed in PBS and incubated with the staining reagents. Zombie Red dye diluted 1:500 in PBS was used to stain cells (15’ minutes at room temperature) followed by staining with antibodies in PBS containing 0.5% BSA and 2 mM EDTA for 30’ at 4°C.

For the staining of intracellular markers with antibodies against FoxP3, cells were fixed and permeabilized using eBioscience™ Foxp3/Transcription Factor Staining Buffer Set (Thermofisher), according to the supplier’s protocol.

Samples were analysed by CytoFLEX S (Beckman Coulter) and data were processed using FlowJo software (FlowJo, LLC, version 10).

Data were analyzed using Prism 7.0 (GraphPad Software, Inc.). Statistical significances data were determined with a regular one-way ANOVA test with Bonferroni post-test correction. Data represent means ± SEM. *P* < 0.05 was considered statistically significant. ^*^= *p* < 0.05, ^**^= *p* < 0.01.

### Immunofluorescence and biodistribution experiments

#### Animal studies

All animal experiments were conducted in accordance with Swiss animal welfare laws and regulations under the license number 27/2015 and 04/2018 granted by the Veterinäramt des Kantons Zürich.

#### Tumor implantation

The murine F9 teratocarcinoma tumor cell line was used to generate the syngeneic tumor model. F9 cells were cultured on 0.1% gelatin-coated tissue culture flasks in DMEM medium supplemented with 10% FCS. F9 tumor cells (25 × 10^6^ cells resuspended in 150 μL of HBSS buffer) were then implanted subcutaneously in the right flank of 129/Sv mice (females, six to eight weeks-old).

### Biodistribution studies

The L19-IL2 immunocytokines (100 μg) were radioiodinated with 125I and Chloramine T hydrate and purified on a PD10 column. Radiolabeled immunocytokines were injected into the lateral tail vein of immunocompetent (129/Sv) mice bearing subcutaneously implanted F9 murine teratocarcinomas. The injected dose per mouse varied between 12 and 15 μg. Mice were sacrificed 24 hours after injection. Organ samples were weighted and radioactivity was counted using a Packard Cobra gamma counter. The protein uptake in the different organs was calculated and expressed as the percentage of the injected dose per gram of tissue (%ID/g). Data were analyzed using Prism 7.0 (GraphPad Software, Inc.). Statistical significances data were determined with a regular one-way ANOVA test with Bonferroni post-test correction. Data represent means ± SEM. *P* < 0.05 was considered statistically significant. ^*^= *p* < 0.05, ^**^= *p* < 0.01, ^***^= *p* < 0.001.

#### Immunofluorescence experiments

Immunofluorescence was performed on frozen murine F9 teratocarcinoma sections (8 μm). The tumor sections were fixed using ice-cold acetone (5 min) and blocked with 20% fetal bovine serum in PBS for 45 min. The L19-IL2 immunocytokines were added to the tumor sections at a concentration of 5 μg/mL in a 2% BSA/PBS solution and incubated for 1 h at room temperature. Anti-human interleukin-2 (final dilution 1:150) (eBioscience, 14-7029-85) was used as the secondary antibody to detect the L19-IL2 immunocytokines. The secondary antibody was added to the tumor sections in a 2% BSA/PBS solution and incubated at room temperature for 1 h. Donkey anti-rat Alexa 488 antibody (final dilution 1:500) (Invitrogen, A21208) was used as the tertiary antibody. Nuclei were counterstained with DAPI. Slides were analyzed with an Axioskop2 mot plus microscope.

### Therapy studies in CT26 colon carcinoma, F9 teratocarcinoma and WEHI-164 fibrosarcoma models

#### Therapy in CT26

Six to eight-weeks-old female BALB/c mice were purchased from Janvier. After one week of acclimatization, mice were injected with 30 × 10^6^ exponentially growing CT26 cells subcutaneously in the right flank. Mice were monitored daily, tumor volume was measured with a caliper and the volume was calculated as follows: (length [mm] × width [mm]^2^)/2. When tumors reached a suitable size (70–100 mm^3^), mice were randomized and injected three times, every 48 hours, into the lateral tail vein with either the immunocytokines (50 μg L19-IL2, 82.5 μg L19L19-IL2) or the saline solution as negative control. Euthanization was performed when the tumor volume reached more than 1500 mm^3^, weight loss exceeded 15%, tumors were ulcerated or 24 hours after every protein injection for infiltrate and cytokine plasma level analysis. Results are expressed as tumor volume in mm^3^ +/− SEM. 5 mice per group were used.

#### Therapy in F9

Six to eight-weeks-old female 129/Sv mice were purchased from Janvier. After 1 week of acclimatization, 6 × 10^6^ exponentially growing F9 teratocarcinoma cells were implanted subcutaneously in the right flank of the mice. Mice were monitored daily, tumor volume was measured with a caliper and the volume was calculated as follows: (length [mm] × width [mm]^2^)/2. When tumors reached a suitable size (70–100 mm^3^), mice were randomized and injected three times, every 48 hours, into the lateral tail vein with either the immunocytokines (100 μg L19-IL2, 165 μg L19L19-IL2) or the saline solution as negative control. Euthanization was performed when the tumor volume reached more than 1500 mm^3^, weight loss exceeded 15%, tumors were ulcerated or 24 hours after every protein injection for infiltrate and cytokine plasma level analysis. Complete remissions were observed until 35 days after therapy start. Results are expressed as tumor volume in mm^3^ +/− SEM. 5 mice per group were used.

#### Therapy in WEHI-164

Six to eight-weeks-old female BALB/c mice were purchased from Janvier. After one week of acclimatization, mice were injected with 3 × 10^6^ exponentially growing WEHI-164 cells subcutaneously in the right flank. Mice were monitored daily, tumor volume was measured with a caliper and the volume was calculated as follows: (length [mm] × width [mm]^2^)/2. When tumors reached a suitable size (about 80 mm^3^), mice were randomized and injected three times, every 48 hours, into the lateral tail vein with either the immunocytokines (100 μg L19-IL2, 165 μg L19L19-IL2), the recombinant human IL2 (40 μg), the anti-PD-1 (clone J43; BioXCell) (200 μg), the combination of L19L19-IL2 and anti-PD-1 (165 μg + 200 μg) or the saline solution as negative control. Euthanization was performed when the tumor volume reached more than 1500 mm^3^, weight loss exceeded 15%, tumors were ulcerated or 24 hours after the last protein injection for infiltrate and cytokine plasma level analysis. Complete remissions were observed until 43 days after therapy start. Results are expressed as tumor volume in mm^3^ +/− SEM. 5 mice per group were used.

#### Cytokine analysis

For the cytokine level analysis, mice were injected according to the therapy schedule and blood was collected 24 hours after the first, second and third immunocytokine injection cycle. Blood was collected rapidly into heparin coated tubes (BD Microtainer, 365966), centrifuged for 10 minutes at 4°C and plasma was collected and stored at −80°C. Samples were sent to Cytolab (Regensdorf) for cytokine plasma level analysis.

#### Analysis of immune infiltrates

Fluorophore-conjugated antibodies against CD3 (clone 17A2), CD4 (clone GK1.5), CD8 (clone 53-6.7), NK1.1 (clone PK136), I-A/I-E (clone M5/114.15.2), CD62L (clone MEL-14), CD44 (clone IM7), PD-1 (clone RMP1-30), TIM3 (clone RMT3-23) and FoxP3 (clone MF-14) and Zombie Red viability dies were all purchased from BioLegend. AH1-loaded, PE-conjugated H-2L^d^ tetramers were obtained as previously described [51].

Tumors, right axillary and right inguinal tumor-draining lymph nodes of mice bearing WEHI-164 fibrosarcoma were collected between 48 and 72 hours after the last injection of fusion proteins or the saline. Tumors were chopped into small pieces and incubated in an orbital shaker at 37°C for 30’ in RPMI-1640 medium containing 1× Antibiotic-Antimycotic (Thermofisher, 15240062), 1 mg/mL Collagenase II (Thermofisher, 17101015) and 0.1 mg/mL DNAse I (Roche, 10104159001). The resulting cells suspensions were filtered through a 70 μm cell strainer (Corning) and treated with Red Blood Cells Lysis buffer (Biolegend, 420301) following supplier’s recommendations. Lymph nodes were smashed on a 70 μm cell strainer (Corning).

Tumor and lymph nodes cells suspension were washed in PBS and incubated with the staining reagents. Zombie Red dye diluted 1:500 in PBS was used to stain cells (15’ minutes at room temperature) followed by staining with antibodies and tetramers in PBS containing 0.5% BSA and 2 mM EDTA for 30’ at 4°C.

For the staining of intracellular markers by means of antibodies against FoxP3, cells were fixed and permeabilized using eBioscience™ Foxp3/Transcription Factor Staining Buffer Set (Thermofisher), according to the supplier’s protocol.

Samples were analysed by CytoFLEX S (Beckman Coulter) and data were processed using FlowJo software (FlowJo, LLC, version 10). The total number of living cells in the tumor was calculated by detracting dead cells and debris by the total number of recorded events.

Data were analyzed using Prism 7.0 (GraphPad Software, Inc.). Statistical significances were determined with a regular one-way ANOVA test with Bonferroni post-test correction. Data represent means ± SEM. *P* < 0.05 was considered statistically significant. ^*^= *p* < 0.05, ^**^= *p* < 0.01.

## SUPPLEMENTARY MATERIALS



## Abbreviations

Amp: Ampicillin
Bp: Base pair
BSA: Bovin serum albumin
CDR: Complementarity determining region
C_H_: Constant domain of antibody heavy chain
CHO: Chines hamster ovary
C_L_: Constant domain of antibody light chain
CV: Column volume
DAPI: 4’,6-diamidin-2-fenilindolo
DMEM: Dulbecco’s Modified Eagle Medium
dNTP: Deoxynucleotides (dATP, dCTP, dGTP, dTTP)
DNA: Deoxyribonucleic acid
E. coli: Escherichia coli
EDA: Extra domain A
EDB: Extra domain B
EDTA: Ethylene diamine immunoabsorbent assay
ELISA: Enzyme-linked immunosorbent assay
FACS: Fluorescence-activated cell sorting
FBS: Fetal bovin serum
FCS: Fetal calf serum
FPLC: Fast protein liquid chromatography
HRP: Horseradish peroxidase
Glu: Glucose
ID/g: Injected dose per gram
IL2: Interleukin 2
Ig: Immunoglobulin
K_D_: Dissociation constant
kDa: Kilo Dalton
LB: Loading buffer
mAb: Monoclonal antibody
M: Mol/l
MES: 2-(N-morpholino)ethanesulfonic acid Buffer
MOPS: 3-(N-morpholino)propansulfonic acid Buffer
MPBS: Skimmed milk powder in PBS
Mw: Molecular weight
OD: Optical density
PBMC: Peripheral blood mononuclear cell
PBS: Phosphate buffer saline
PCR: Polymerase chain reaction
PD-1: Programmed cell death protein 1
PD-L1: Programmed death-ligand 1
PEG Polyethilene glycol; PEI: Polyethileneimine
pI: Isoelectric point
Rpm: Revolutions per minute
RPMI: Roswell Park Memorial Institute
RT: Room temperature (20–24°C)
RU: Resonance units
scFv: Single chain variable fragments
SDS-PAGE: Sodium dodecylsulfate-polyacrilamide gel electrophoresis
SEM: Standard error of the mean
SPR: Surface plasmon resonance
T_1/2_: Half-life
TBE: Tris/Borate/EDTA buffer
TEA: Triethylamin
TGE: Transient gene expression
TIM: T-cell immunoglobulin and mucin domain 1
TNF: Tumor necrosis factor
Tris: Tris (hydroxymethyl)aminomethane
V_H_: Variable domain of antibody heavy chain
V_L_: Variable domain of antibody light chain
