# Supplementary material for: A novel format for recombinant antibody-interleukin-2 fusion proteins exhibits superior tumor-targeting properties in vivo
Source: Oncotarget. 2020 Oct 13;11(41):3698–711. doi: 10.18632/oncotarget.27726 (PMC7566808; doi:10.18632/oncotarget.27726)
Supplement: Supplementary file 1 [file oncotarget-11-3698-s001.pdf]

A novel format for recombinant antibody-interleukin-2 fusion proteins exhibits superior tumor-targeting properties *in vivo*

SUPPLEMENTARY MATERIALS

A

Protein	Average Yield (mg/L production)
L19-IL2 (various VH-VL linkers)	~ 10
L19L19-IL2	~ 4
IL2-L19L19-IL2	~ 3
L19-IL2-L19	~9
IL2-L19L19	~4

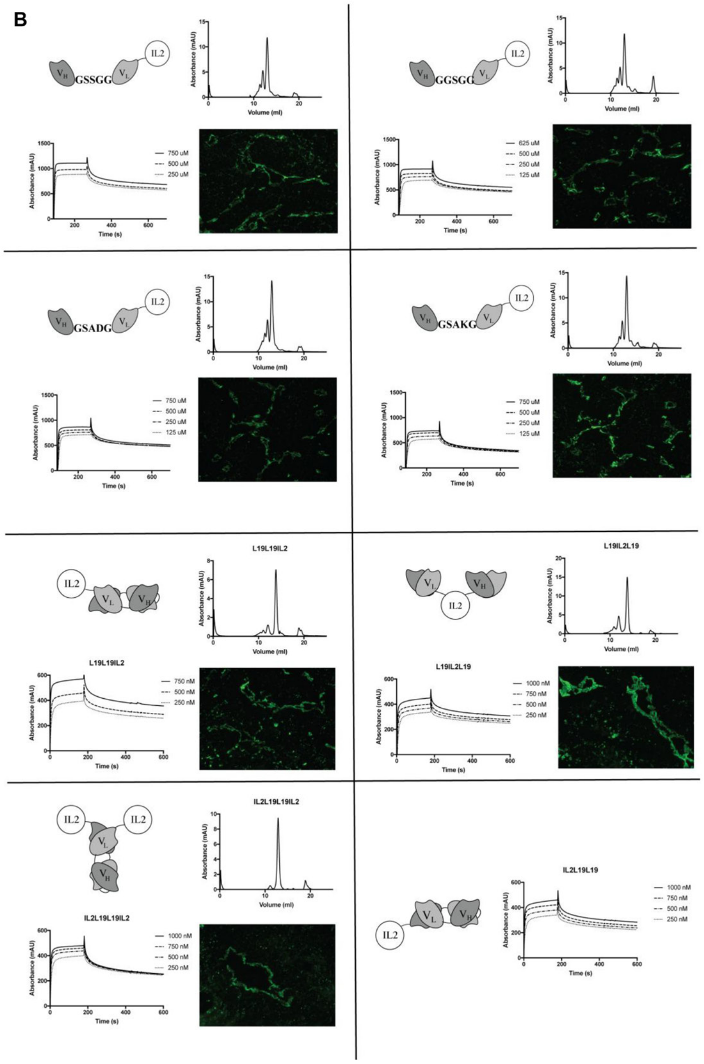

Supplementary Figure 1: (**A**) Average production yields. (**B**) *In vitro* analysis of the new fusion proteins. Size exclusion chromatography was performed on a S200 increase column, the main peak in the graphs represent the monomeric proteins. The BIAcore analysis was performed on a 7B89 coated CM5 and proteins were injected at concentrations between 1000 nM and 125 nM. The immunofluorescences were performed on F9 teratocarcinoma tumor slices. All the fusion proteins displayed nice perivascular targeting properties.

A

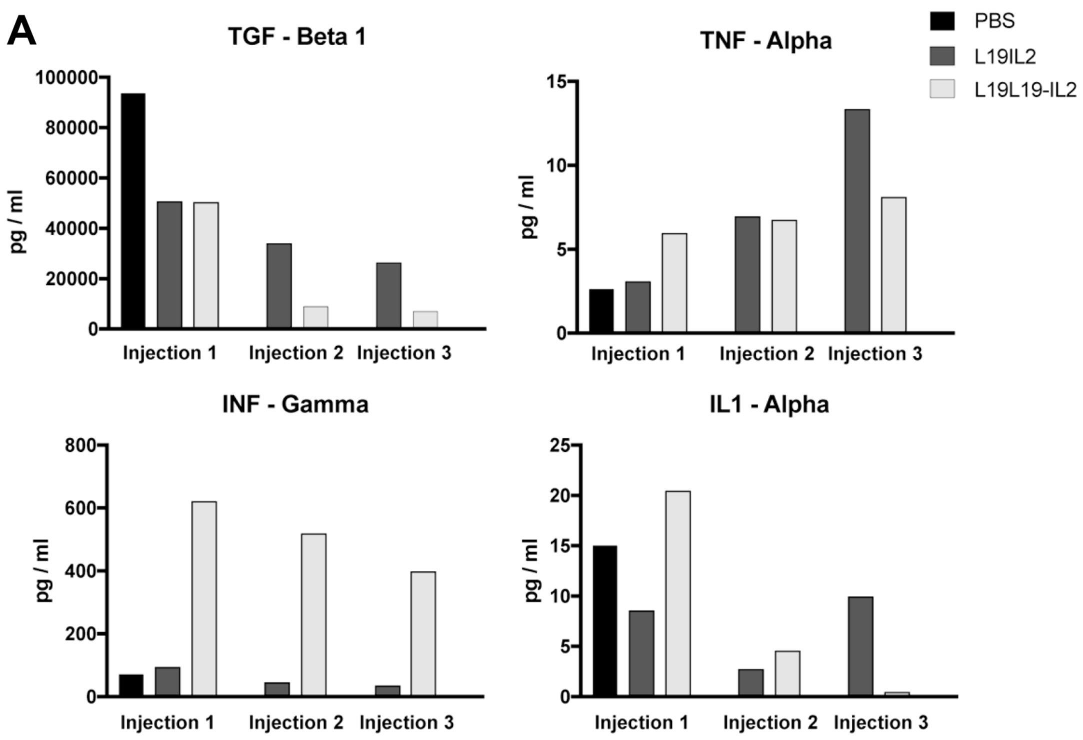

B

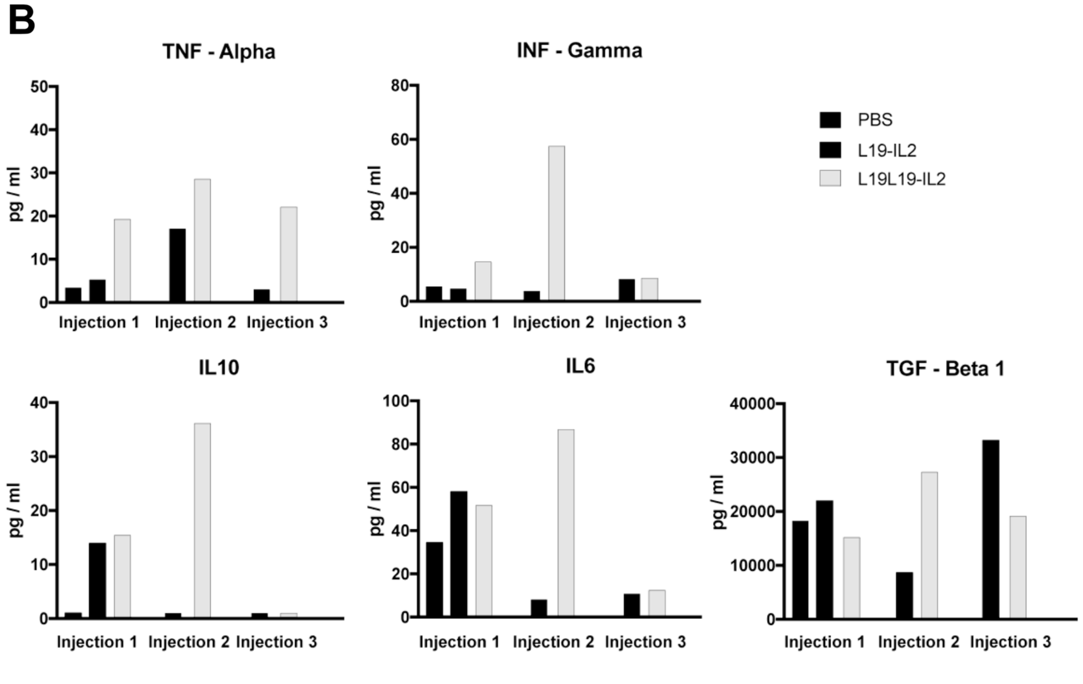

Supplementary Figure 2: (**A**) Cytokine analysis performed on plasma collected from treated CT26 bearing mice 24 hours after the last injection cycle. (**B**) Cytokine analysis performed on plasma collected from treated F9 bearing mice 24 hours after the last injection cycle.

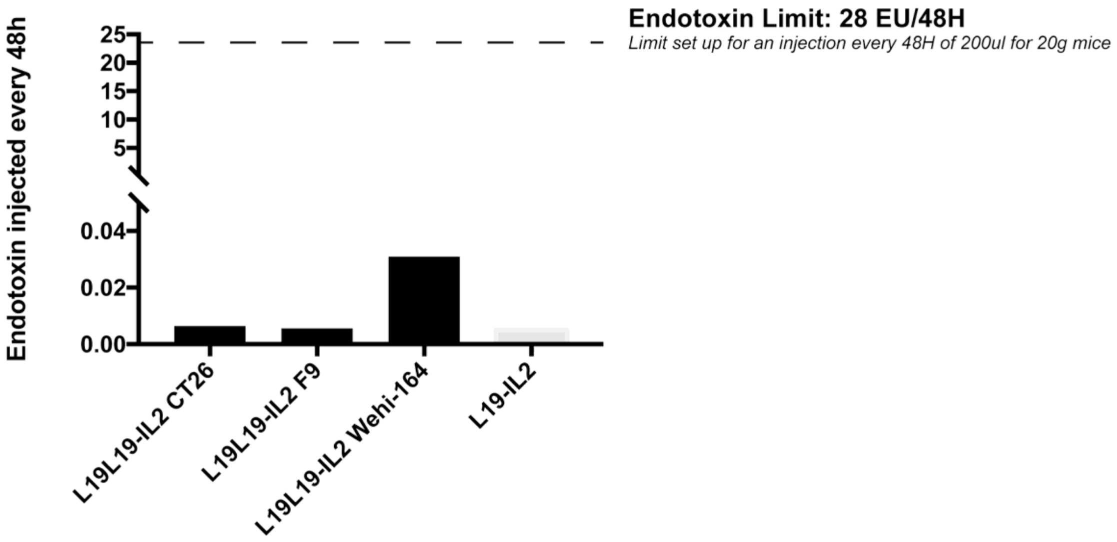

Supplementary Figure 3: Endotoxin levels of proteins used for the therapies in CT26, F9 and Wehi-164 models.

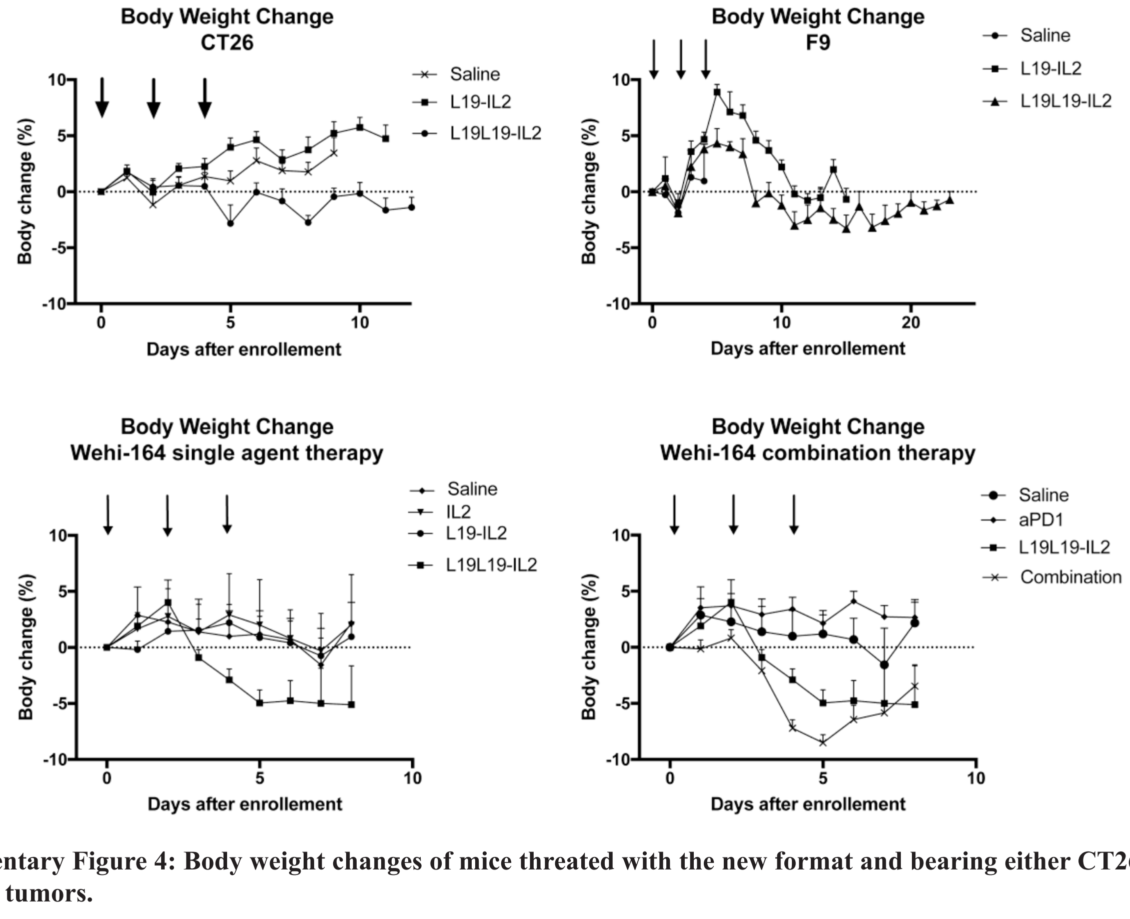

Supplementary Figure 4: Body weight changes of mice threated with the new format and bearing either CT26, F9 or Wehi-164 tumors.

Supplementary Table 1A: Amino acid sequence of L19

**VH**
EVQLLESGGGLVQPGGSLRLSCAASGFTFSSFSMSWVRQAPGKGLEWVSSISGSSGTTYYADSVK<br>GRFTISRDNSKNTLYLQMNSLRAEDTAVYYCAKPFPYFDYWGQGTLVTVSS
**VL**
EIVLTQSPGTLSLSPGERATLSCRASQSVSSSFLAWYQQKPGQAPRLLIYYASSRATGIPDRFSGSGSGTDF<br>TLTISRLEPEDFAVYYCQQTGRIPPTFGQGTKVEIK

Supplementary Table 1B: Amino acid sequence of IL2

APTSSSTKKTQLQLEHLLLDLQMILNGINNYKNPKLTRMLTFKFYMPKKATELKHLQCLEEELKPLEEV<br>LNLAQSKNFHLRPRDLISNINVIVLELKGSETTFM<br>CEYADETATIVEFLNRWITFCQSIISTLT

Supplementary Table 2A: Primers used for the cloning of L19 diabody-IL2 conjugate with GSSGG VH-VL domain linker sequence

HindIIISIP	CCCAAGCTTGTCGACCATGGGCTGGAGCC
L19Linker	GAGCCGGAAGAGCTACTACCCGATGAGGAA<br>GATTTGATTTCCACCTTGGTCCCTTG
LinkerIL2	TCGGGTAGTAGCTCTTCCGGCTCATCGTCCAG<br>CGGCGCACCTACTTCAAGTTCTACA
IL2stopNotI	TTTTCCTTTTGCGGCCGCTCATTAAGTCAGTGTTGAGATGAT

Supplementary Table 2B: Primers used for the cloning of L19 diabody-IL2 conjugates with different VH-VL domain linker sequences

**Fragment A**		
**Clone**	**Forward Primer**	**Backward Primer**
GGSGG	TAATACGACTCACTATAGGG	CACCGCCTGATCCCCCACTCGAGACGGTGACCA<br>GGGT
GSADG	TAATACGACTCACTATAGGG	CGTCTGCTGACCCACTCGAGACGGTGACCAGGGTT<br>CCC
GSAKG	TAATACGACTCACTATAGGG	ACCTTTTGCTGACCCACTCGAGACGGTGACCAGGGT<br>TCCC
**Fragment B**		
**Clone**	**Forward Primer**	**Backward Primer**
GGSGG	TCTCGAGTGGGGGATCAGGCGG<br>TGAAATTGTGTTGACGCAG	TAGAAGGCACAGTCGAGG
GSADG	CCGTCTCGAGTGGGTCAGCAGAC<br>GGTGAAATTGTGTTGACGCAGTCT	TAGAAGGCACAGTCGAGG
GSAKG	CCGTCTCGAGTGGGTCAGCAAAAG<br>GTGAAATTGTGTTGACGCAGTCT	TAGAAGGCACAGTCGAGG

Supplementary Table 2C: Primers used for the cloning of new formats of L19-IL2 immunocytokine

NheLead>	CTAGCTAGCTAGGACCATGGGCTGGAGCCTGATCCTCCTGTTCCTCGTCGCTGTGGCTA
HindLead>	CCCAAGCTTGGGGACCATGGGCTGGAGCCTGATCCTCCTGTTCCTCGTCGCTGTGGCTA
IL2G4S3<	CCGCCAGAACCCCCTCCGCCTGACCCGCCTCCACCAGTCAGTGTTGAGATGATGCTTTG
G4S3L19>	GGTCAGGCGGAGGGGGTTCTGGCGGTGGCGGATCGGAGGTGCAGCTGTTGGAGTCTGGG
L19Hind<	GAAGCTTCCTTTGATTTCCACCTTGGTCCCTTG
LnkDP47>	ATGGAGCAGGTGGCAGTGCAGGAGCGGACGGGGGTGAGGTGCAGCTGTTGGAGTCTGGG
HindLnk>	CAAGCTTGGATGGAGCAGGTGGCAGTGCAGGAG
G4S3IL2>	GGTCAGGCGGAGGGGGTTCTGGCGGTGGCGGATCGGCACCTACTTCAAGTTCTACAAAG
L19G4S3<	CCGCCAGAACCCCCTCCGCCTGACCCGCCTCCACCTTTGATTTCCACCTTGGTCCCTTG
IL2G4S3Bam<	CGCGGATCCCCCTCCGCCTGACCCGCCTCCACCAGTCAGTGTTGAGATGATGCTTTG
BamG4S3L19>	CGCGGATCCGGCGGTGGCGGATCGGAGGTGCAGCTGTTGGAGTCTGGG
L19StopNot<	TTTTCCTTTTGCGGCCGCTCATTATTTGATTTCCACCTTGGTCCCTTG
IL2StopNot<	TTTTCCTTTTGCGGCCGCTCATTAAGTCAGTGTTGAGATGATGCTTTG
DP47G4S2.5<	CCCTGACCCTCCGCCACCAGAGCCCCCACCTCCACTCGAGACGGTGACCAGGGTTCC
G4S2.5VL>	GGGCTCTGGTGGCGGAGGGTCAGGGGGAGGCGGTGAAATTGTGTTGACGCAGTCTCCA
LeadIL2>	CTGTTCCTCGTCGCTGTGGCTACAGGTGTGCACTCGGCACCTACTTCAAGTTCTACAAA

Supplementary Table 3A: Biodistribution statistical analysis with a regular one-way ANOVA test with Bonferroni post-test correction

Bonferroni's multiple comparisons test	Mean Diff.	95.00% CI of diff.	Significant?	Summary	Adjusted *P* Value	E-?		
L19L19IL2 vs. GSSGG	2,77	0.4486 to 5.091	Yes	*	0,0137	A		
L19L19IL2 vs. GGSGG	2,17	–0.1514 to 4.491	No	ns	0,0767	B		
L19L19IL2 vs. GSADG	3,22	0.8986 to 5.541	Yes	**	0,0037	C		
L19L19IL2 vs. GSAKG	2,61	0.2886 to 4.931	Yes	*	0,0218	D		
L19L19IL2 vs. IL2L19L19	3,88	1.559 to 6.201	Yes	***	0,0006	F		
L19L19IL2 vs. IL2L19L19IL2	3,99	1.669 to 6.311	Yes	***	0,0004	G		
L19L19IL2 vs. L19IL2L19	2,44	0.1186 to 4.761	Yes	*	0,0356	H		
Test details	Mean 1	Mean 2	Mean Diff.	SE of diff.	n1	n2	t	DF
L19L19IL2 vs. GSSGG	7,81	5,04	2,77	0,7652	5	3	3,62	18
L19L19IL2 vs. GGSGG	7,81	5,64	2,17	0,7652	5	3	2,836	18
L19L19IL2 vs. GSADG	7,81	4,59	3,22	0,7652	5	3	4,208	18
L19L19IL2 vs. GSAKG	7,81	5,2	2,61	0,7652	5	3	3,411	18
L19L19IL2 vs. IL2L19L19	7,81	3,93	3,88	0,7652	5	3	5,07	18
L19L19IL2 vs. IL2L19L19IL2	7,81	3,82	3,99	0,7652	5	3	5,214	18
L19L19IL2 vs. L19IL2L19	7,81	5,37	2,44	0,7652	5	3	3,189	18

$P < 0.05$ was considered statistically significant. * = $p < 0.05$, ** = $p < 0.01$, *** = $p < 0.001$.

Supplementary Table 3B: Tumor-to-organ ratios of the evaluated candidates

	Tumor	Liver	Lung	Spleen	Heart	Kidney	Intestine	Tail	Blood
Candidate GSSGG %ID/g	5,037375	0,190482	0,380072	0,30724	0,160539	0,341311	0,590734	0,385654	0,697588
Tumor-to-Organ Ratio		26,4454	13,25373	16,39557	31,37799	14,75889	8,527317	13,06191	7,221137
	Tumor	Liver	Lung	Spleen	Heart	Kidney	Intestine	Tail	Blood
Candidate GGSGG %ID/g	5,639988	0,200776	0,525106	0,363241	0,272429	0,38189	0,87176	0,489171	0,88337
Tumor-to-Organ Ratio		28,09093	10,74066	15,52684	20,70258	14,76862	6,469657	11,52968	6,38463
	Tumor	Liver	Lung	Spleen	Heart	Kidney	Intestine	Tail	Blood
Candidate GSAKG %ID/g	5,203951	0,153771	0,314178	0,277318	0,135841	0,262445	0,604225	0,246137	0,400286
Tumor-to-Organ Ratio		33,84216	16,56372	18,76526	38,30904	19,82875	8,612604	21,1425	13,00058
	Tumor	Liver	Lung	Spleen	Heart	Kidney	Intestine	Tail	Blood
Candidate GSADG %ID/g	4,590503	0,165467	0,686017	0,277419	0,124334	0,276858	0,547397	0,507652	0,431101
Tumor-to-Organ Ratio		27,74274	6,691527	16,54718	36,92086	16,58072	8,386057	9,042625	10,64832
	Tumor	Liver	Lung	Spleen	Heart	Kidney	Intestine	Tail	Blood
Candidate L19L19-IL2 %ID/g	7,810526	0,214871	0,705788	0,418478	0,220676	0,430225	1,395944	0,581245	0,602738
Tumor-to-Organ Ratio		36,3499	11,0664	18,66411	35,39359	18,15452	5,595158	13,43759	12,95841
	Tumor	Liver	Lung	Spleen	Heart	Kidney	Intestine	Tail	Blood
Candidate L19-IL2-L19 %ID/g	5,367861	0,359649	0,796827	0,438532	0,292274	0,584234	0,940613	0,448973	0,709335
Tumor-to-Organ Ratio		14,92529	6,736546	12,24052	18,36587	9,187863	5,706769	11,95586	7,567457
	Tumor	Liver	Lung	Spleen	Heart	Kidney	Intestine	Tail	Blood
Candidate IL2-L19L19-IL2 %ID/g	3,825273	0,125816	1,656943	0,185343	0,085798	0,20342	0,509177	0,380887	0,26553
Tumor-to-Organ Ratio		30,40365	2,308633	20,6389	44,58469	18,80483	7,512654	10,04308	14,4062
	Tumor	Liver	Lung	Spleen	Heart	Kidney	Intestine	Tail	Blood
Candidate IL2-L19L19 %ID/g	3,938794	0,187694	0,342869	0,285979	0,150536	0,338655	0,936547	0,492622	0,472035
Tumor-to-Organ Ratio		20,98518	11,48775	13,77301	26,16518	11,6307	4,205655	7,995564	8,344279
